# In Vitro and Ectopic In Vivo Studies toward the Utilization of *Rapidly* Isolated Human Nasal Chondrocytes for Single-Stage Arthroscopic Cartilage Regeneration Therapy

**DOI:** 10.3390/ijms23136900

**Published:** 2022-06-21

**Authors:** Gyözö Lehoczky, Raluca Elena Trofin, Queralt Vallmajo-Martin, Shikha Chawla, Karoliina Pelttari, Marcus Mumme, Martin Haug, Christian Egloff, Marcel Jakob, Martin Ehrbar, Ivan Martin, Andrea Barbero

**Affiliations:** 1 Department of Orthopaedic Surgery and Traumatology, University Hospital of Basel, 4031 Basel, Switzerland; gyoezoe.lehoczky@unibas.ch (G.L.); marcus.mumme@ukbb.ch (M.M.); christian.egloff@usb.ch (C.E.); 2 Department of Biomedicine, Tissue Engineering Laboratory, University Hospital Basel, University of Basel, 4031 Basel, Switzerland; ralucaelena.trofin@usb.ch (R.E.T.); chawla.shikha16@gmail.com (S.C.); karoliina.pelttari@usb.ch (K.P.); andrea.barbero@usb.ch (A.B.); 3 Department of Obstetrics, University Hospital Zurich, University of Zurich, 8091 Zurich, Switzerland; queralt.vallmajomartin@usz.ch (Q.V.-M.); martin.ehrbar@usz.ch (M.E.); 4 Department of Orthopaedic Surgery, University Children’s Hospital of Basel, 4056 Basel, Switzerland; 5 Department of Plastic, Reconstructive and Aesthetic Surgery and Hand Surgery, University Hospital of Basel, 4031 Basel, Switzerland; martin.haug@usb.ch; 6 CrossKlinik, 4054 Basel, Switzerland; marcel.jakob@unibas.ch

**Keywords:** cartilage regeneration, autologous chondrocyte implantation, nasal chondrocytes, single-stage, arthroscopy, tissue engineering, polyethylene glycol, hydrogel, platelet lysate

## Abstract

Nasal chondrocytes (NCs) have a higher and more reproducible chondrogenic capacity than articular chondrocytes, and the engineered cartilage tissue they generate in vitro has been demonstrated to be safe in clinical applications. Here, we aimed at determining the feasibility for a single-stage application of NCs for cartilage regeneration under minimally invasive settings. In particular, we assessed whether NCs isolated using a short collagenase digestion protocol retain their potential to proliferate and chondro-differentiate within an injectable, swiftly cross-linked and matrix-metalloproteinase (MMP)-degradable polyethylene glycol (PEG) gel enriched with human platelet lysate (hPL). NC-hPL-PEG gels were additionally tested for their capacity to generate cartilage tissue in vivo and to integrate into cartilage/bone compartments of human osteochondral plugs upon ectopic subcutaneous implantation into nude mice. NCs isolated with a *rapid* protocol and embedded in PEG gels with hPL at low cell density were capable of efficiently proliferating and of generating tissue rich in glycosaminoglycans and collagen II. NC-hPL-PEG gels developed into hyaline-like cartilage tissues upon ectopic in vivo implantation and integrated with surrounding native cartilage and bone tissues. The delivery of NCs in PEG gels containing hPL is a feasible strategy for cartilage repair and now requires further validation in orthotopic in vivo models.

## 1. Introduction

Articular cartilage has a limited intrinsic capacity to heal, and therefore, cartilage defects if left untreated can lead to the early onset of degenerative changes. Commonly used treatment options, including marrow stimulation techniques and the implantation of autologous osteochondral plugs, are associated with a variable quality of the repair tissue and a limited reproducibility of the clinical outcome [1]. Thus, cell-based therapies have been introduced to obtain better clinical results, using autologous cells to create tissue-engineered implantable grafts [2]. Currently, articular chondrocyte (AC)-based regeneration procedures—ACI (autologous chondrocyte implantation)—are well-established cartilage-repair techniques that can lead to long-lasting clinical improvement in specific cases [3,4,5]. However, they still have important drawbacks, as we will discuss later on in this section. 

Recent cartilage repair techniques based on the grafting of cells of a different origin than ACs (e.g., naive or ex vivo cultured mesenchymal stromal/stem cells from adipose tissue, bone marrow or synovium) are reported to induce positive clinical outcomes [6,7], but still, the absence of consolidated protocols for the preparation and application modalities of such cellular grafts hamper their wide clinical use for the management of cartilage lesions. A variety of other approaches using cell-free materials, mostly in order to enhance cartilage regeneration through the stimulation of bone-marrow-derived cells, have also been described [8]. However, the clinical outcomes are very heterogenous and long-term data are lacking. 

ACI can result in durable hyaline-like cartilage in relatively young patients [3,4], but suffers from the following drawbacks: (1) donor site morbidity caused by harvesting of cartilage biopsy from a healthy compartment of the damaged joint [9], (2) unpredictable variability in the chondrogenic capacity of articular chondrocytes (ACs) from different donors [10], (3) the need for two operations of the joint at two different timepoints, and (4) high costs associated with the in vitro culture of the chondrocytes [11]. A possible solution for overcoming the first two drawbacks might consist of the use of nasoseptal chondrocytes (NCs), instead of ACs, as a cell source. Nasoseptal cartilage biopsies can be harvested under local anesthesia, with minimal donor site morbidity [12] and with a standard procedure, allowing the perichondrium, i.e., tissue known to actively contribute to the formation of neocartilage in the place of the induced lesion, to be kept in situ [13,14,15]. Isolated NCs have been shown to exhibit a reproducible, cartilage-forming capacity, even if harvested from elderly individuals [16,17]. Moreover, the feasibility and safety of using autologous NCs (in the form of engineered cartilage tissues) for the repair of articular cartilage lesions have been recently demonstrated [18], and a phase II multicenter clinical trial to investigate the efficacy of this strategy (BIOCHIP, http://biochip-h2020.eu, accessed on 17 May 2022), coordinated by the University Hospital of Basel, is ongoing.

To overcome the other aforementioned drawbacks, NCs should be used immediately after their isolation from the cartilage biopsy, and possibly be embedded in a gel that allows their grafting in the joint through a minimally invasive approach. For such a single-stage setting—i.e., nasoseptal cartilage harvesting and use for implantation in the next hours without expansion of the cells—an enzymatic cartilage digestion protocol much shorter than the typical one, which lasts 12–22 h [10,19,20,21], is required. Vedicherla and Buckley [22] have demonstrated that a very short exposure (i.e., 1 h) of *bovine* nasal cartilage samples with a high concentration of type II collagenase (i.e., 3000 U/mL) was effective for retrieving cells at a similar quantity to that generated using a typical 12 h digestion protocol with a lower concentration of type II collagenase (i.e., 750 U/mL). This rapid protocol even resulted in superior cell viability after digestion. Additionally, the authors report that cells isolated with such *rapid* protocols exhibited a higher propensity to proliferate and to produce a cartilage matrix in alginate. However, such investigations were performed using cells after an expansion phase in monolayer and seeding at a relatively high density (i.e., 4 × 10^6^ cells/mL), i.e., in conditions not resembling a single-stage scenario in which only low numbers of native cells are available. Thus, whether freshly isolated human NCs using a rapid protocol can efficiently proliferate and chondro-differentiate once encapsulated in a gel at a low (clinically relevant) cell density, remains an open question. 

Different types of hydrogels have been used as carrier systems to grow chondrocytic cells and to deliver them in vivo, due to their high water content and physiologically-relevant characteristics. Up until now, mostly natural hydrogels, derived from extracellular matrix macromolecules such as collagen, fibrin, hyaluronic acid, or chitosan among others, have been employed. Even though natural materials are biocompatible and promote cell growth, they possess an inherent batch-to-batch variability and a risk of contamination. Therefore, synthetic hydrogels that mimic the native microenvironment and are inherently inactive as well as highly reproducible have been developed [23]. Enzymatically cross-linked polyethylene glycol (PEG) hydrogels (i.e., with activated transglutaminase coagulation factor XIIIa) have been shown to be able to support cell growth in numerous studies. The incorporation of both appropriate cell adhesion sites and protease sensitive linkers (for biodegradability) allows for in vivo remodeling [24,25,26,27]. Thus, PEG hydrogels represent suitable scaffolds for articular cartilage repair. Recently, we have shown that expanded human NCs embedded in PEG hydrogels at a high density (20–25 × 10^6^ cells/mL) containing Bone Morphogenetic Protein-2 (BMP-2) were capable of forming hyaline cartilage tissue in subcutaneous pockets of nude mice [28]. However, in clinically compatible, single-stage scenarios, freshly isolated NCs should populate hydrogels only at low-density, and in this setting, a single growth factor would likely not be sufficient to stimulate both proliferation and cartilage matrix deposition. Multiple growth factors would be available by using human platelet lysate (hPL), a pooled, platelet-rich plasma (PRP) extract. This extract is a safe and cost-effective source of growth factors including platelet-derived growth factor (PDGF), transforming growth factor (TGF), vascular endothelial growth factor (VEGF) and fibroblast growth factor (FGF), among others [29]. It has previously been shown that hPL efficiently promotes proliferation and cartilage matrix deposition by mesenchymal stromal cells (MSCs) [30,31,32] and chondrocytes [31,33]. It also enhances the proliferation and chondrogenic capacity of MSCs once combined into hydrogels [34]. 

The final goal of this study is to implement the use of autologous NCs in a single-stage, minimally invasive technique for the repair of small to middle-size focal cartilage lesions (2–5 cm^2^). Here, specific investigations were performed with the aim to assess whether:

(1) *Rapid* isolation of chondrocytes from human nasoseptal cartilage (i.e., 4 h vs. 22 h treatment with type II collagenase) yields comparable numbers of viable cells capable of proliferating and generating hyaline-like cartilage *in vitro*,
(2) *rapidly* isolated, non-expanded NCs embedded at low density in optimized PEG hydrogels enriched with human PL (NC-hPL-PEG gel) are able to proliferate and produce a cartilaginous matrix in vitro and *in vivo*, and
(3) NC-hPL-PEG gels are capable to efficiently integrate with osteochondral tissues (obtained from allogenic knee joints) in an *in vivo* environment.

Considering our intention of obtaining initial preclinical data on the performance of *rapidly* isolated NCs from cartilage of *human* origin, for the two latter aforementioned aims (2 and 3), we used two ectopic *in vivo* models widely used to assess the cartilage regenerative properties of cell-based grafts.

## 2. Results

### 2.1. Cell Yields after Rapid Digestion of Nasoseptal Cartilage Biopsies

**Figure 1:**
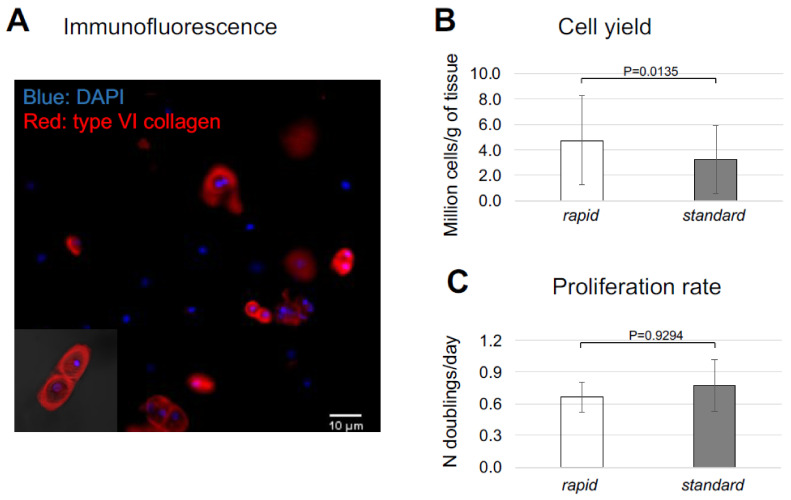
**Properties of nasoseptal chondrocytes (NCs) generated after the *rapid* isolation protocol.** Nasoseptal cartilage from human patients was digested 22 h with 0.15% type II collagenase (*standard* protocol) or 4 h with 1.5% type II collagenase (*rapid* protocol). (**A**) Immunofluorescence picture of cells isolated after the *rapid* isolation protocol (red: type VI collagen, blue: DAPI). (**B**) Cell yield (i.e., millions of cells/g of tissue, N = 51). (**C**) Proliferation rate (i.e., number of population doublings/day) of cells expanded in monolayer (N = 11). Values represent mean ± SD of results.

Immunohistofluorescence staining for type VI collagen, a marker characteristic of the cartilage pericellular matrix, revealed the presence of large fractions of chondrons in the cell suspension generated after the *rapid* digestion protocol of nasoseptal cartilage biopsies (Figure 1A). As compared to the *standard* digestion protocol, the *rapid* isolation protocol yielded higher cell numbers (3.3 × 10^6^ ± 2.7 × 10^6^ vs. 4.8 × 10^6^ ± 3.5 × 10^6^ cells/gram of tissue, *p =* 0.0135, N = 51 paired samples) (Figure 1B). In contrast, the viability of cells isolated with the two isolation protocols was comparable (99.0 ± 1.3% vs. 98.4 ± 1.6%, respectively, N = 11 paired samples).

### 2.2. Proliferation and Cartilage-Forming Capacity of Nasoseptal Chondrocytes (NCs) Isolated by Rapid Digestion

**Figure 2:**
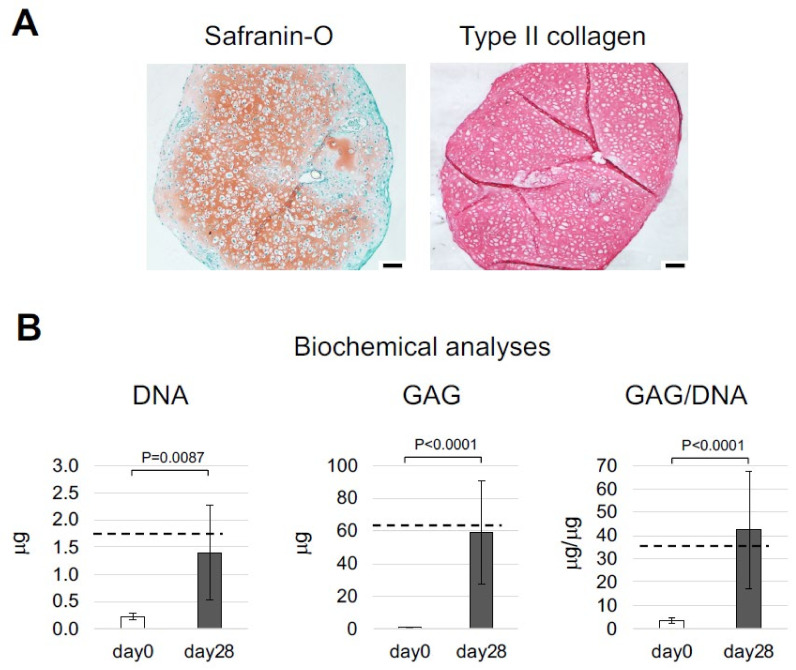
**Chondrogenic capacity of nasoseptal chondrocytes (NCs) generated after the *rapid* isolation protocol.** Freshly isolated NCs were cultured in pellets in medium containing human platelet lysate (hPL) for 28 days. (**A**) Representative Safranin-O and type II collagen staining of resulting cartilaginous tissues. Scale bars = 100 µm. (**B**) Glycosaminoglycans (GAG), DNA and GAG/DNA contents of generated cartilaginous tissues. Values are mean ± SD of results of samples generated with cells from seven different donors. Dash lines represent the mean values of the parameters measured in control pellets generated by passage 2 standard isolated NCs at day 28.

Cells isolated after the digestion of nasoseptal cartilage biopsies were either plated in culture dishes and expanded for two passages to estimate their proliferation capacity and post-expansion differentiation capacity, or centrifuged to generate pellets that were cultured in different media, i.e., hPL-ChM (i.e., the medium used also for the culture of NCs in PEG, medium components detailed in the “Materials and Methods” Section 4.3.2) and SFM-ChM (medium normally used to assess the chondrogenic capacity of cells [10], see its composition in Section 4.3.2). The proliferation rate of NCs isolated with the *rapid* digestion protocol was similar to that of cells isolated with the *standard* digestion protocol (0.66 ± 0.14 and 0.78 ± 0.25 doublings/day, respectively) (Figure 1C). Histological and immunohistochemical analyses of pellets generated using *rapidly* isolated, non-expanded NCs demonstrate that the cartilaginous proteins glycosaminoglycan (GAG) and type II collagen (Col II) were highly present and uniformly distributed in the tissues (Figure 2A), and that type VI collagen (Col VI) was localized in pericellular regions (Supplementary Figure S2). Biochemical analyses demonstrate that *rapidly* isolated, non-expanded NCs cultured for 28 days in hPL-ChM increased in number and accumulated large amounts of GAGs, as demonstrated by the 2.6- and 27.5-fold increase of the DNA and GAG contents, respectively, in pellets. The GAG/DNA contents of pellets were 3.3 ± 1.2 and 42.4 ± 25.1, respectively, at day 0 and day 28 (Figure 2B). A similar histological quality and similar levels of GAG/DNA were observed in pellets generated by *rapidly* isolated, non-expanded NCs in SFM-ChM (Supplementary Figure S1). Control pellets generated by P2-expanded NCs that were isolated with the *standard* digestion protocol (i.e., conditions normally used to assess chondrogenic capacity of cells [10]) increased their DNA and GAG contents during 28 days of culture in SFM-ChM to a similar extent as the non-expanded, *rapidly* isolated NCs (Figure 2B, dash lines). 

Overall, this first set of results demonstrates that the *rapid* isolation of human nasoseptal cartilage yields a high number of viable cells capable of proliferating and generating hyaline-like cartilage. Moreover, we show that hPL is a supplement capable of efficiently promoting the proliferation and differentiation of NCs. This mixture of growth factors was thus used for the following experiments in which NCs were cultured in PEG gels.

### 2.3. Proliferation and Cartilage-Forming Capacity of NCs Isolated with the Rapid Digestion Protocol in PEG Gels Containing hPL (hPL-PEG Gels)

To exclude the possibility that the addition of hPL in PEG hydrogels alters the mechanical properties, we first performed rheometric analyses of PEG hydrogels either containing or not containing 5% (*v*/*v*) hPL. The results show that the hydrogels had a similar storage modulus (265.6 ± 38.1 Pa vs. 232.4 ± 38.8 Pa) and loss modulus (0.298 ± 0.043 Pa vs. 0.306 ± 0.106 Pa) in the presence or absence of hPL (Supplementary Figure S3A,B).

**Figure 3:**
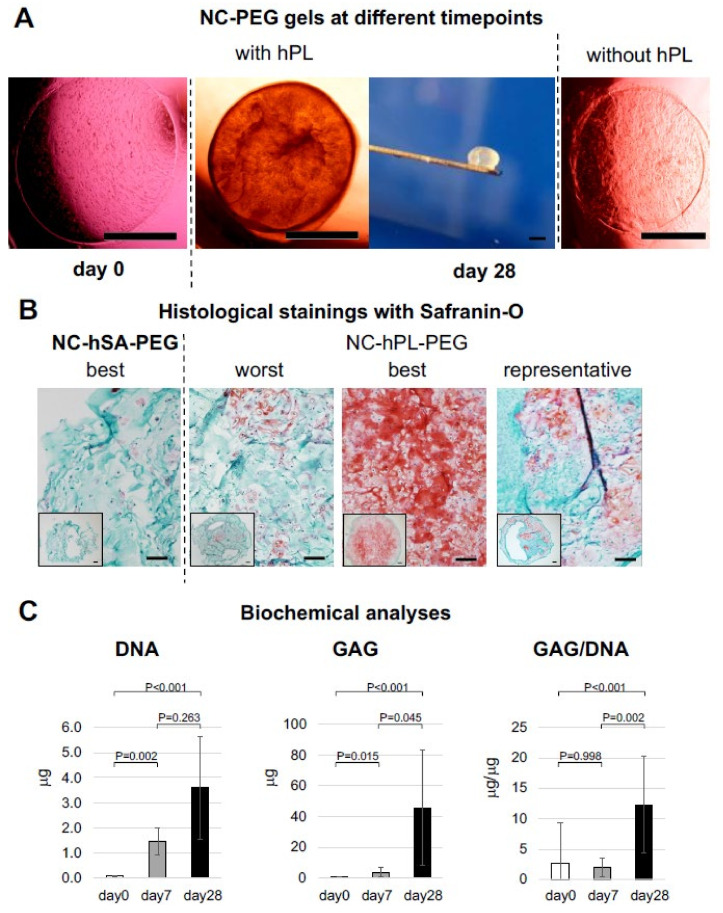
**Histological characterization of the constructs generated by culturing nasoseptal chondrocytes (NC) in Polyethylene glycol (PEG) gel (NC-PEG).** Constructs were generated by culturing *rapidly* isolated NCs in PEG gel in medium containing human serum albumin (NC-hSA-PEG, as control) or human platelet lysate (NC-hPL-PEG, see Materials and Methods for details). (**A**) Macroscopic appearances of the constructs during the in vitro cultivation. Scale bar = 1 mm. (**B**) Safranin-O staining of a control NC-hSA-PEG (I) construct as well as worst (II), best (III) and representative (IV) quality NC-hPL-PEG constructs generated after 28 days in culture (Bern score values of the corresponding samples are 3.5, 4.5, 8.5, 5.5). Scale bars = 100 µm. The inserts are low magnification images of the entire constructs. (**C**) Glycosaminoglycans (GAG), DNA and GAG/DNA contents of constructs generated at day 0, 7 and 28, respectively. Values are mean ± SD of results of samples generated with cells from nine different donors.

NCs generated after the *rapid* isolation protocol were then immediately embedded in PEG hydrogels either containing 5% *v*/*v* hPL (NC-hPL-PEG) or not (as control, NC-hSA-PEG) at the clinically relevant density of 6000 cells per 20 μL gels, and cultured for up to 28 days. Macroscopic examination showed that the initially transparent and discoidal NC-hPL-PEG gels developed into dense spheroidal structures that were whitish in color, indicating extensive matrix deposition by the embedded NCs (Figure 3A). In contrast, control gels cultured without hPL (Figure 3A) remained transparent, their density did not significantly increase, and their shape did not round into a spheroid during the entire culture time.

Histological analyses demonstrated that *rapidly* isolated NCs cultured in hPL-PEG produced cartilage-specific proteins. At the end of the culture, highly cellular gels positively stained for GAGs could be generated, even if at variable amounts and uniformity depending on the NC donor (see “worst”, “best” and “representative” pictures in Figure 3B). In contrast, cell proliferation and cartilage matrix deposition by NCs in control hSA-PEG gels was not observed. Indeed, the majority of the control samples (approx. 70%) disaggregated during the culture, and thus could not be processed histologically or biochemically (Figure 3B shows the best generated NC-hSA-PEG sample with a Bern score of 1.5).

Safranin-O-stained images of gels generated from *rapidly* isolated, non-expanded NCs after 21 and 28 days of culture were graded using the established Bern score system [35] (see Section 4.5.1). We obtained the following main results: (i) the total Bern score of NC-hPL-PEG gels cultured for 21 days was significantly higher than that of NC-hSA-PEG gels, (ii) intensity of staining and cell morphology were the categories that most differed between these two gels, and (iii) no significant improvement in total Bern score, intensity of staining and cell morphology was observed in NC-hPL-PEG cultured for one additional week (21 vs. 28 days) (Table 1).

Biochemical analyses of gels indicated that the DNA contents of NC-hPL-PEG gels significantly increased after 7 days of culture (by 27.7-fold, *p =* 0.002) and further increased in the subsequent three weeks (by 3.1-fold, *p =* 0.263). We estimated cell numbers in gel constructs based on the corresponding DNA content (using the factor 7.7 pg DNA/cell [36]), and derived that in the first 7 days of culture, NCs underwent 4.62 ± 0.89 population doublings, while during the entire culture time (28 days) cells underwent 6.33 ± 0.51 population doublings in total. GAG content, in contrast, was still relatively low (3.47 ± 3.0 µg per gel construct) at 7 days of culture but significantly increased to up to 110 µg/gel (range: 28.0–110.9 µg) at 28 days. No significant differences in GAG/DNA content were observed between day 0 and day 7, whereas a significant increase of GAG/DNA ratio was measured between 7 and 28 days (2.0 ± 1.5 vs. 12.3 ± 7.9, *p =* 0.002) (Figure 3C).

**Figure 4:**
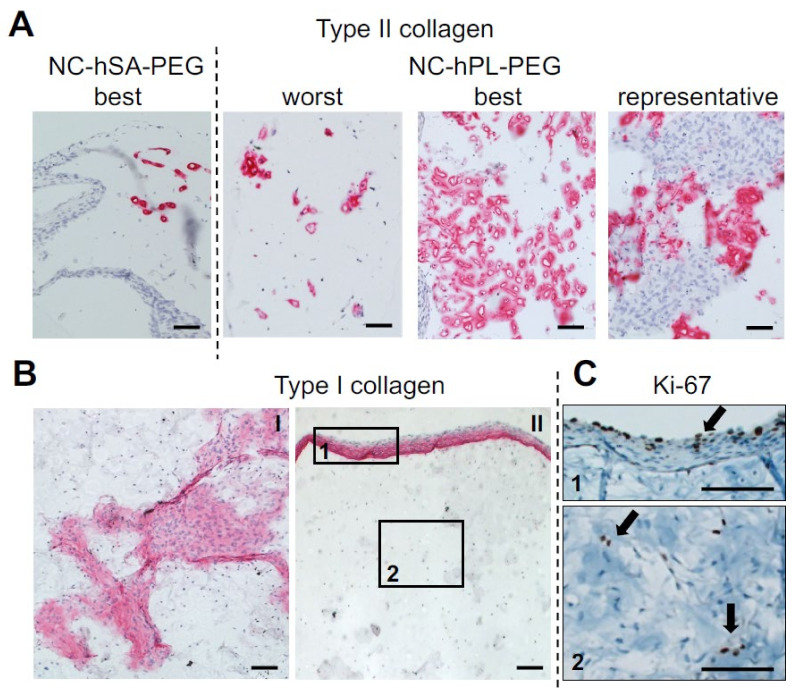
**Immunohistochemical characterization of the constructs generated by culturing nasoseptal chondrocytes (NC) in Polyethylene glycol (PEG) gel (NC-PEG).** Constructs were generated by culturing *rapidly* isolated NCs in PEG gel in medium containing human serum albumin (NC-hSA-PEG, control) or platelet lysate (NC-PL-PEG) (see Materials and Methods for details). (**A**) Type II collagen staining of worst, best and representative quality constructs. Scale bar = 100 µm. (**B**) Type I collagen staining in central (I) and peripheral (II) areas of NC-hPL-PEG constructs. (**C**) Ki-67 staining of NC-hPL-PEG constructs. Images were taken from the same sample as image B/II, of regions (squares) “1” and “2”. Scale bars = 100 µm. Black arrows indicate Ki-67 positive cells.

Immunohistological characterization of NC-hPL-PEG demonstrated that Col II was mainly localized in areas with round chondrocyte-like cells (Figure 4A), while Col I was mainly localized in areas containing cells that had fibroblastic appearances, localized at the surface or within the gels (Figure 4B). These Col-I-producing cells could not yet be fully differentiated NCs, rather than contaminant perichondral cells, considering the care used to eliminate any residue of perichondral tissue from the collected nasoseptal cartilage biopsies (see Section 4.2). In contrast, Col VI staining was scarcely present at pericellular regions of the NC-hPL-PEG gels (Supplementary Figure S2), indicating an ongoing phase of remodeling the pericellular matrix (PCM). Ki-67-positive cells were mainly localized at the surface of the gels (in the Col-I-positive areas). A few single cells positive for Ki-67 and with round morphology could be observed in Col-I-negative areas (Figure 4C). 

Overall, these results indicate that NCs generated using the *rapid* isolation protocol, once cultured at low density in PEG hydrogels, efficiently proliferate and accumulate cartilage matrix components, but only in the presence of hPL.

### 2.4. In Vivo Cartilage Forming Capacity of NC-PL-PEG Gels

**Figure 5:**
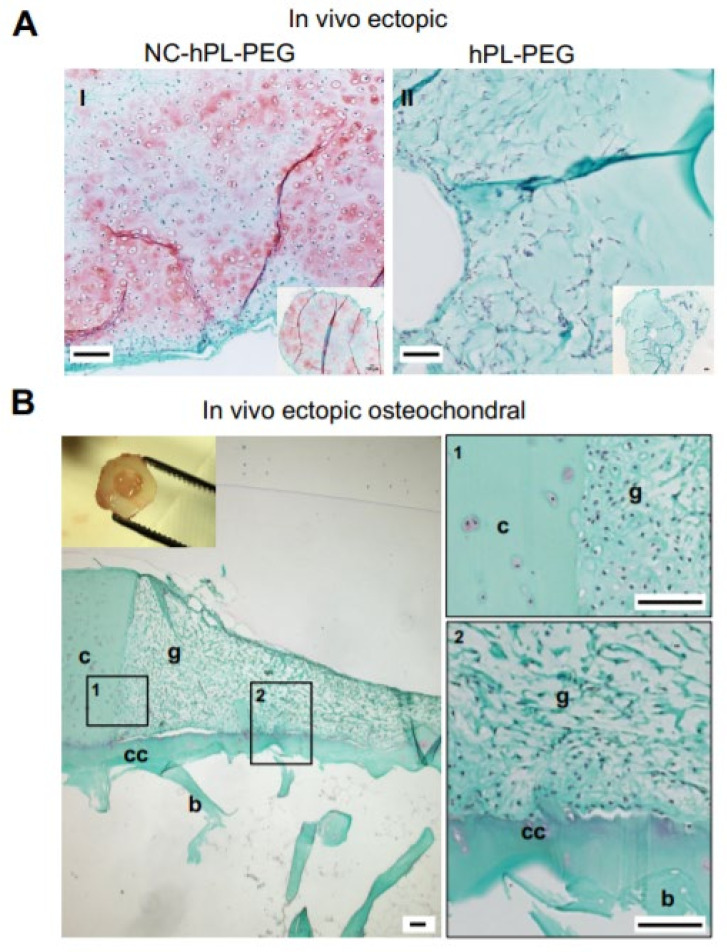
**Cartilage forming capacity and integration capacity of the constructs in vivo.** Platelet-lysate-containing (hPL-PEG) gels with or without (as control) nasoseptal chondrocytes were implanted in the subcutaneous pocket of nude mice, without preculturing, alone (ectopic, **A**) or injected into a cartilage defect generated in a human osteochondral tissue explant (ectopic human osteochondral model, (**B**) (see Materials and Methods for details). (**A**) Safranin-O staining of resulting cell-based (**I**) and cell-free (**II**) constructs. Scale bars = 100 µm. The insert is a low magnification image of the entire construct. (**B**) Safranin-O staining of explants. The inset shows the combined construct at the time of explantation. Images “1” and “2” are high magnification pictures of areas “1” and “2” on the low magnification image (on the left). Scale bars = 100 µm; c = native cartilage, b = native bone, cc = native calcified cartilage, g = NC-hPL-PEG graft.

**Figure 6:**
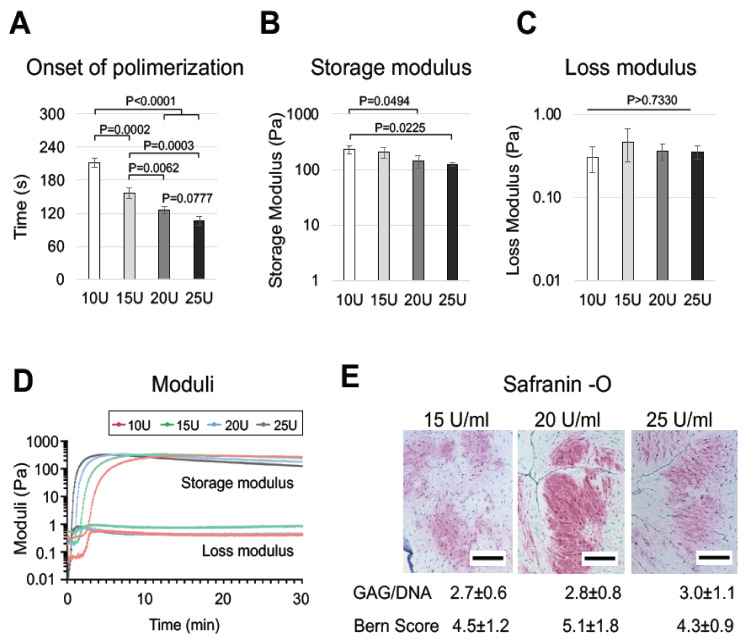
**Optimization of PEG hydrogel constructs for clinical use.** (**A**–**C**) Generation kinetics of 1.5% PEG gels with different concentrations of factor XIII used for polymerization (10, 15, 20 and 25 U/mL): onset of gelation (**A**), storage (**B**) and loss (**C**) modulus, generation kinetics curve of storage and loss moduli (**D**). (**E**) Safranin-O staining of constructs generated using 15, 20 and 25 U/mL of factor XIII, GAG/DNA contents and Bern score grading of the corresponding samples. Values are mean ± SD of results of three replicate samples. Scale bars = 100 µm.

The former in vitro studies demonstrate that hPL is essential for promoting proliferation and cartilage matrix deposition by NCs seeded at low density in PEG hydrogels. We thus assessed whether NC-hPL-PEG gels are also capable of efficiently developing into cartilaginous tissues once transplanted in ectopic subcutaneous pockets of nude mice, i.e., a permissive but inert environment for chondrogenesis. Histological analyses of the explanted NC-hPL-PEG gels, after 28 days in vivo without in vitro pre-culture, demonstrate the presence of high cell numbers and variable amounts of cartilaginous matrix in the tissues (Figure 5A). Grading of Safranin-O stained slides shows that the total Bern score, intensity of staining and cell morphology of the in vivo NC-hPL-PEG were comparable to the in vitro analogues (Table 1). Acellular PEG (negative control, hPL-PEG) was partially invaded by inflammatory/fibroblastic murine cells (Figure 6A), and no sign of cartilage tissue was observed (Bern score = 0). Immunostaining results demonstrate the abundant presence of Col VI in pericellular regions of the NC-hPL-PEG, but an absence of this PCM-specific component in the control hPL-PEG gels (Supplementary Figure S2).

To assess the capacity of NC-hPL-PEG gel to integrate with native cartilage/subchondral bone tissues, it was grafted into defects created in osteochondral (OC) plugs (obtained from allogenic knee joints), and further implanted, without pre-culturing, in ectopic pockets of nude mice. As shown in Figure 5B, the newly formed tissue in the OC grafts adhered well to the surrounding cartilage and to the calcified cartilage/subchondral bone. Cells with round morphology, surrounded by a matrix faintly stained by Safranin-O, were present in the gel at the border to native cartilage tissue. In contrast, in the central part of the OC tissue, cells with a fibroblastic appearance and matrix with limited/null GAG were present.

To better appreciate the performance of the NC-hPL-PEG gel in the OC tissues, we scored the histological quality of the explants (*n* = 9 explants of three donors), taking into account the following three categories: integration with cartilage (min: 0, max: 3), integration with bone (min: 0, max: 3) and quality in the tissue distant from the margins (min: 0, max: 3). The observed total score of 5.3 ± 1.5 (range: 3–8) indicated a certain variability in the performance of NC-hPL-PEG in such a model. Regarding the integrative properties of NC-hPL-PEG, the score in the category “integration with cartilage” was higher (2.4 ± 0.8) and less variable than that in the category “integration with bone” (1.8 ± 1.1) or the overall tissue quality (1.4 ± 1.0, Table 2).

### 2.5. Optimization of NC-hPL-PEG Gels toward Clinical Use

With the aim of the clinical translation of NC-hPL-PEG gel, the manufacturing process should be developed to be more cost-effective and time-effective. We thus sought to (i) simplify the hydrogel formulation, keeping only the minimal components for successful NC performance, and (ii) achieve faster gelation times to meet in situ surgery requirements (i.e., gelation time below 2 min).

Several studies have shown that the role of RGD peptide (Arginyl-glycyl-aspartic acid cell attachment-site) could be fulfilled by other ECM-derived components whose structures contain the RGD motif (such as fibronectin or vitronectin, among others), without having a negative influence on the embedded cells’ functions [37]. Human PL, already added in the constructs, is a rich source of growth factors and bioactive molecules, among which also fibronectin is found. Therefore, by adding hPL in the formulation, we might ensure that the cell adhesion and cell spreading functions are fulfilled, without the need for an external, covalently bound RGD peptide motif. We first assessed the mechanical properties of hPL-PEG gels either containing or not containing RGD, and demonstrated that both the storage and loss moduli were similar in hPL-PEG gels containing or not containing the covalently bound RGD adhesion peptide (Supplementary Figure S3C,D). We then characterized the proliferation and chondrogenic capacity of NCs embedded in hPL-PEG hydrogel either containing or not containing RGD. Biochemical analyses of the generated constructs show similar amounts of GAG (9.11 ± 1.16 vs. 7.98 ± 1.78) and DNA (3.07 ± 0.50 vs. 2.38 ± 0.37) in gels containing or not containing RGD.

Next, we intended to optimize the gelation time to enable faster in situ hydrogel polymerization into the defect site. Hydrogel gelation time was drastically shortened (from 3.52 ± 0.12 to 1.77 ± 0.12 min) by increasing the concentration of factor XIII from 10 U/mL to 25 U/mL (Figure 6A). In addition to a significant reduction of gelation time, hydrogels polymerized with increasing factor XIII concentrations featured significantly reduced storage moduli (from 232.4 ± 38.75 Pa to 124.1 ± 8.731 Pa, Figure 6B,D), while they maintained similar loss moduli (Figure 6C,D).

Histological and biochemical analyses of the constructs generated with different amounts of factor XIII showed a similar extent of chondrogenesis (Figure 6E). Therefore, the MMP-degradable hPL-PEG gel presented herein, further developed to contain a minimal number of components and to have a gelation time matching clinical requirements, was suitable for chondrogenic differentiation of *rapidly* digested, non-expanded NCs.

## 3. Discussion

We demonstrated that a *rapid* digestion protocol of human nasoseptal cartilage biopsies (i.e., 4 h incubation with 1.5% collagenase type II) as compared to a *standard* digestion protocol (i.e., 22 h incubation with 0.15% collagenase type II) yielded a higher number of viable cells with similarly high proliferation and cartilage-forming capacities. Once embedded in MMP-degradable PEG gel containing human platelet lysate (hPL) at relatively low cell density (i.e., 300,000 cells/mL), *rapidly* isolated nasoseptal chondrocytes (NCs) were capable of efficiently proliferating and generating in vitro and in vivo hyaline-like cartilage that integrated well with surrounding native cartilage and bone tissues. 

In a previous study, Vedicherla and Buckley [22] investigated the performance of nasal chondrocytes isolated from *bovine* nasal cartilage using short or long (i.e., 1 h or 12 h) incubation with low or high concentrations of type II collagenase (i.e., 750 or 3000 U/mL collagenase II, corresponding to 0.4% or 1.6%, respectively). The least (i.e., 1 h incubation with 750 U/mL) and the most (i.e., 12 h incubation with 3000 U/mL) aggressive protocols were suboptimal due to incomplete tissue digestion or relatively high cell mortality, respectively. The other digestion protocols (1 h incubation with 3000 U/mL and 12 h incubation with 750 U/mL, comparable to the ones used in our study) yielded similar numbers of cells (1–1.5 × 10^6^ cells/gram of tissues). Cell yields in our study were higher (4.8 × 10^6^ ± 3.5 × 10^6^ and 3.3 × 10^6^ ± 2.7 × 10^6^, respectively, for the rapid and standard isolation protocols). These differences in cell yields can be due to the different species used in the two studies (bovine vs. human) or to a limited amount of post-digestion processing performed in our study (i.e., no filtering of the tissue/cell suspensions and one instead of three washes) that could have resulted in the loss of a certain fraction of the isolated cells. In our study, we did not compare the cartilage forming capacity of freshly isolated NCs with the *rapid* vs. *standard* isolation protocol due to the limited size of the samples obtained from the patients, and due to our envisioned clinical application (i.e., use of NCs in a single-stage cartilage repair setting). However, results from the previously mentioned work and other studies [38,39] using different types of cartilage (i.e., articular cartilage, nucleus pulposus and annulus fibrosus) clearly demonstrate the superior cartilage-forming capacity of *rapidly* isolated cartilage cells as compared to those isolated with long digestion protocols. The superior performance of cells generated with a rapid isolation protocol might be due to a preservation of the native pericellular matrix (PCM), a narrow ECM surrounding the chondrocytes that functions as transducer of both biomechanical and biochemical signals for the chondrocytes [40]. In particular, in the abovementioned studies, chondrocytes remained, surrounded by a matrix intensely stained for type VI collagen, one of the main components of the pericellular matrix, which is known to have a key role in cartilage homeostasis [41]. In accordance with these findings, we also observed a high amount of PCM surrounding the rapidly isolated NCs. Immunohistochemical analyses of Col VI demonstrated a variable degree of PCM remodeling in the different models used. The higher abundance of Col VI in NC-pellets, as compared to NC-hPL-PEG gels, can be explained by the fact that, in the former model, NCs are mainly induced to produce cartilage matrix, while in the hPL-PEG, the cells are initially stimulated to proliferate (and thus to remodel the initially present pericellular matrix) and then to synthesize new PCM. More Col-VI-positive matrix is present in the in vivo (vs. in vitro) NC-hPL-PEG constructs, probably due to the fact that under the subcutaneous pocket of nude mice, the ECM remodeling and, consequently, the production of new PCM is faster/more efficient. 

It is important to consider that NCs could acquire an “unstable” phenotype as a consequence of the harsh enzymatic treatment. However, our results generated in vitro and in vivo, as well as the results reported in other studies [22,42,43,44], suggest a good capacity of the cells to recover after this initial stress.

In order to test the feasibility of using *rapidly* isolated NCs from an initial small cartilage biopsy (as described by Mumme et al. [18]) for the repair of articular cartilage defects, we tested their cartilage-forming capacity once embedded at low density in PEG gels. The PEG gel has a well-known potential for allowing a seamless modification of its physical and biological properties, as well as its components ratio. These features enable PEG hydrogels to have a great advantage over naturally occurring biomaterials, since they can be tailored and optimized to the needs of the implantation site [25,27,45]. For example, the increase in initial PEG polymer concentration will enable the formation of stiffer hydrogels. Additionally, the variation of the built-in proteolytic sites can be designed to be degradable with lower or higher efficiency [46,47]. In this study, we decided to use PEG hydrogels fulfilling minimal criteria for the growth of cells in 3D, namely the presence of the MMP degradable site *GPQGIWGQ* in the backbone of the hydrogel and the cell adhesion site RGD [23], contained in the supplemented hPL. Using the recently described seamless integration of other building blocks, such as growth factors, growth factor binding sites or ECM components, offers the possibility of creating a more supportive initial environment for the growth of chondrocytes, or even guiding their differentiation [48,49,50].

We observed that, in the absence of bioactive supplements, NCs remained low in number and deposited limited/no matrix in PEG gels, thus indicating that under our experimental conditions, the maintenance of the pericellular matrix was not sufficient for promoting efficient proliferation and matrix deposition by the *rapidly* isolated NCs. In the previously mentioned studies, *rapidly* isolated chondrocytes were able to increase their initial number and accumulate cartilage matrix in alginate beads. However, in these studies, cells were seeded at a density 10-fold higher than the one used in our study (4 × 10^6^ vs. 0.3 × 10^6^) and cultured in medium containing 10% FBS [38] or in defined medium containing Transforming Growth Factor (TGF) beta [22]. These supplements could raise difficulties for the GMP-compliant manufacturing of the gel. To address the latter issue and towards clinical adoption, we decided to use a clinical-grade human platelet lysate (hPL, at 5% *v*/*v*) as a growth factor mixture to promote cell proliferation and matrix deposition. The hPL is considered a safe and cost-effective source of bioactive molecules such as coagulation factors, adhesion molecules, proteoglycans, growth factors (e.g., PDGF, TGF, VEGF, FGF) and anti-inflammatory cytokines [29,33], and it has been shown to promote the proliferation and differentiation of different chondrogenic cells [51]. In addition, hPL has previously been incorporated in different hydrogels (e.g., based on dextran-tyramine [34] or hyaluronic acid [52]). In the first study mentioned, Moreira Teixeira et al. demonstrated that hPL incorporated in dextran-tyramine hydrogels enhanced the migration of human chondrocytes and mesenchymal stromal cells (hMSCs) as well as the proliferation and chondrogenic (but not osteogenic and adipogenic) capacity of the incorporated hMSCs. In the second study, Jooybar et al. showed that hPL incorporated in hyaluronic acid hydrogels enhanced the metabolic activities and the chondrogenic potential of the incorporated hMSCs. However, there are also few reports in the literature demonstrating that some commercially available hPL preparations have been shown to negatively affect the matrix deposition of chondrocytes [53,54]. We selected one hPL preparation (the MultiPL’100i from Macopharma) among others for its capacity to enhance the proliferation capacity of human nasoseptal chondrocytes while maintaining their good post-expansion cartilage-forming capacity. Our results show that *rapidly* isolated NCs cultured in PEG in the presence of the selected hPL efficiently proliferate and produce an abundant GAG and type II collagen matrix. The findings of a more pronounced increase in DNA contents in the first week’s culture and a significant GAG accumulation from day 7 to day 28 suggest that hPL has a biphasic role: it initially promotes proliferation of NCs and later, once NCs reach a sufficient cell density, mainly promotes chondrogenesis. Interestingly, some round cells in the central, type-I-collagen-negative parts of the graft were positive for Ki-67, suggesting that hPL can provide mitogenic signals also to cells with a differentiated appearance. 

Cellular morphology and matrix deposition in the majority of the constructs were not uniform through the hydrogel. In particular, cells with fibroblastic/elongated morphology and ECM poorly/negatively stained for cartilage specific proteins could be observed within the gels even after 28 days of culture. Thus, further optimization of the materials and/or protocols should be introduced in order to achieve uniform chondrogenesis of the NCs. 

The cartilage-forming capacity of the freshly prepared (and not pre-cultured) NC-hPL-PEG gels was additionally tested ectopically in nude mice. We showed that the initial grafts contained sparse NCs developed into highly cellular constructs rich in cartilaginous matrix within only 28 days. In our setting, hPL was not covalently bonded in the PEG gels; thus, our finding demonstrated that key bioactive factors present in the hPL, such as BMP-6 and TGF-β [34], can stay entrapped in the gels at a concentration and can for a certain time be sufficient to trigger NC proliferation and chondrogenesis. We are aware that longer in vivo incubation times will be required to investigate the stability of the cartilage tissues generated with our model, even if we do not expect that our grafts will not undergo the process of endochondral bone formation, considering our previous results indicating that cartilage grafts generated with NCs even after in vitro hypertrophic induction, once implanted in vivo, did remain hyaline-like in nature [55]. Such investigations, together with a tumorigenic study, will be conducted in a future study to assess the safety and feasibility of our approach towards clinical implementation. To mimic our envisioned approach for the single-stage repair of cartilage defects, we finally injected the NC-hPL-PEG gels into an osteochondral defect created from human joint samples that were further grafted ectopically in nude mice. The demonstrated graft–cartilage integration could have been mediated by the NCs embedded in the PL-PEG gel and/or the articular chondrocytes derived from the margin of the cartilage. The hPL incorporated in the PEG gel could have indeed promoted the migration/invasion of chondrocytes, as this growth factor mixture was shown to enhance the chemo-attractant properties when primary chondrocytes were used in an in vitro migration test [34]. The finding that the central region of the osteochondral defect was rich in cells with a fibroblastic appearance (thus suggesting an effective proliferation of the initially low number of grafted NCs), with limited cartilaginous matrix, is likely related to the reduced time of observation after implantation. Additional in vivo studies including different time points will be required to evaluate the kinetics of NC proliferation, matrix deposition and integration processes. Finally, in our study, graft–bone integration was rather variable, probably due to irregular amounts of hyaline/calcified cartilage tissues remaining in the osteochondral defects after their debridement with the standard ring curette [56]. In this context, the utilization of laser technology [57] to refresh cartilage defects with opto-acoustic feedback [58,59], instead of the classical surgical instrumentation used in this paper, could allow the generation of more uniform defects to test the integration properties of a cell-loaded gel at more standardized conditions in future studies.

In our envisioned strategy, the patient would be exposed to a first surgical intervention under local anesthesia for the removal of the nasoseptal cartilage biopsy. The patient would then be monitored for a certain time to exclude complications related to this surgery and finally be transferred to an orthopaedics surgery department for second surgery (i.e., injection of the cell preparation), under spinal or general anesthesia. The pre-implantation procedures described can be relatively long overall, and thus are compatible with our strategy of generating the therapeutic cells within 4 h. However, to reduce possible downtimes of this procedure, a shorter isolation protocol would be preferable. Additional experiments will then be required to assess the performance of *human nasal* chondrocytes isolated with shorter protocols (i.e., 45–60 min) previously used to isolate *bovine nasal* chondrocytes [22], *goat articular* chondrocytes [43] or *human articular chondrocytes* [42,44]. Even if the collection of the nasal cartilage biopsy could be counted as an additional intervention, we decided to classify this strategy as a “single-stage procedure”, similarly to other previously published procedures. In such single-stage procedures, first harvesting from a different compartment than the knee (e.g., the iliac crest) was performed to collect cells for transplantation (i.e., bone marrow cells), that were implanted in the knee of the patient, within a second operation, and still called single-stage surgery [42,44,60]. Our procedure, in line with the other, aforementioned ones, is different from the classical “two-stage” procedures in which an in vitro culture step of the harvested cells (after harvesting/first operation) is required to generate the therapeutic cell preparation that will be used in a later, second intervention to repair the cartilage defect.

We are aware that a possible problem associated with our proposed approach is represented by the required involvement of rhinoplasty surgeons for the collection of the nasoseptal cartilage biopsy, which might increase the logistic expense and complexity of the therapeutic approach. However, this procedure, if properly organized, is feasible, as demonstrated in our recently performed clinical studies in which (i) stromal vascular fraction cells or (ii) human nasoseptal chondrocytes (harvested by plastic surgeons from fat tissue and nasal cartilage biopsies, respectively) were implanted into bone or cartilage defects by orthopedic/trauma surgeons, respectively [18,61].

In conclusion, we have demonstrated that rapidly isolated nasal chondrocytes from the nasal septum, once embedded at a low density relevant for clinical use and without expansion, in PEG gels containing hPL can efficiently proliferate and produce hyaline cartilage tissue in vitro and in vivo. Moreover, we have provided evidence of the efficiency of NC-hPL-PEG gels in integrating with surrounding native joint tissues. Future proof-of-concept studies in a large orthotopic animal model should be performed to assess the feasibility of using the optimized NC-hPL-PEG gel for the repair of cartilage defects. NC-hPL-PEG gel could also be envisioned for the repair of other tissues, e.g., for degenerated intervertebral discs, where protective properties of the pericellular matrix may provide a substantial benefit by initial protection of the implanted cells from the harsh mechanical and inflammatory conditions. Finally, before using this envisioned therapeutic strategy in patients, further necessary actions must be carried out to obtain the approval of the modified PEG gel as medical device or together with nasoseptal chondrocytes as a “combination medical device–advanced therapy medicinal product”.

## 4. Materials and Methods

### 4.1. Collection of Human Nasoseptal Cartilage Specimens

Human nasal septum biopsies were provided by the plastic surgeons of the University Hospital of Basel from patients who underwent rhinoplasty surgeries. All patients gave their written informed consent before operation and harvesting, and use occurred in accordance with the local ethical committee (University Hospital Basel; Prof. Dr. Kummer; approval date 26/03/2007 Ref Number 78/07). Before the intervention, the nose of the patients was carefully disinfected from outside and inside using the Octenisept^®^ antiseptic lotion (Schülke & Mayr GmbH). The harvested nasosaptal cartilage biopsies were transported in a medium containing 100 U/mL penicillin, 100 mg/mL streptomycin. Data of a total of 53 patients were used (26 males and 27 females, mean age 35.3 ± 13.0 years).

### 4.2. Enzymatic Digestion of Human Nasoseptal Septal Cartilage Specimens

Nasoseptal cartilage specimens were carefully inspected for the presence of remnants of perichondrium and in case the perichondrium was clearly present, it was dissected from the nasal cartilage using sterile forceps and scalpel as previously described [62]. The nasoseptal cartilage tissues were then cut into small pieces of less than 1 × 1 mm with a sterile scalpel, washed three times with phosphate buffered solution (PBS) containing 100 U/mL penicillin, 100 mg/mL streptomycin, and processed for the digestion steps. Initial nasoseptal cartilage biopsies were either digested with the previously established protocol (*standard* digestion [10]) or with a novel rapid isolation protocol (*rapid* digestion), as described below. In both cases, cartilage tissues were weighed before being digested.

*Rapid* digestion: Approx. 200 mg cartilage tissue was collected per 1.5 mL vial and incubated at 37 °C for 4 h in 1.5 mL of 1.5% type II collagenase (385 U/mg, Worthington, Biochemical Corp., Lakewood, NJ) in a Basic Medium (BM) supplemented with 5% Fetal Bovine Serum (FBS) (see below for the composition of BM). Tubes were sealed with paraffin and placed on an orbital shaker at a velocity of 240 rpm. After digestion, the cell suspension was transferred into a 15 mL Falcon tube. The activity of the collagenase was neutralized by adding 10 mL/tube of BM (Dulbecco’s Modified Dulbecco’s modified Eagle’s medium containing 4.5 mg/mL D-glucose, 0.1 mM nonessential amino acids, 1 mM sodium pyruvate, 100 mM HEPES buffer, 100 U/mL penicillin, 100 mg/mL streptomycin, and 0.29 mg/mL L-glutamine) supplemented with 10% FBS. Following centrifugation (3 min/1500 rpm), the cells were resuspended in BM with 10% FBS. Trypan-blue(Sigma-Aldrich)-stained cells were counted to estimate cell yields (i.e., million cells/gram of tissue) and viability (as percentage of viable cells). An aliquot of cell suspension was plated on a glass slide and the cells were processed for immunofluorescence analyses as described below.

*Standard* digestion: cartilage pieces were collected as previously described and incubated at 37 °C for 22 h with 0.15% type II collagenase on an orbital shaker (40 rpm) [10]. After digestion, cells were counted as described above.

### 4.3. Cell Culture

Freshly isolated cells were cultured using the two digestion protocols in monolayer to estimate their proliferation capacity. Culture of NCs in micromass pellets was also performed to investigate their cartilage forming capacity, either after the cartilage digestion step or after the expansion phase. Culture of non-expanded NCs in PEG gels was carried out to assess proliferation and cartilage forming capacity of the cells.

#### 4.3.1. Monolayer Culture 

NCs were expanded in monolayer at a seeding density of 5.000 to 10.000 cells/cm^2^ for two passages (corresponding to 8–10 population doublings) in BM supplemented with 10% FBS, 5 ng/mL Fibroblast Growth Factor 2 (FGF-2, R&D Systems Minneapolis, MN, USA) and 1 ng/mL Transforming Growth Factor β1 (TGF-β1; R&D Systems, Minneapolis, MN, USA) [10]. NCs were cultured in a 100 × 20 mm Petri dish (Falcon^®^ Tissue Culture Dish, Corning Life Sciences, Glendale, AZ, USA, Ref. No. 353003) or in a T150 flask (Falcon^®^ Tissue culture flask, Corning Life Sciences, Glendale, AZ, USA, Ref. No. BDAA353003), respectively for passage 0 -> passage 1 or passage 1- > passage 2. Media changes were performed twice per week. After each passage, cell numbers were counted as described earlier and proliferation rate (i.e., number of population doublings/day) was estimated.

#### 4.3.2. Culture of NCs in Pellets

Chondrogenic differentiation was induced by culturing *rapidly* isolated NCs in micromass pellets, using the following two chondrogenic media (ChM): (i) SFM-ChM (BM supplemented with 1.25 mg/mL human serum albumin (CSL Behring, Bern, Switzerland, 43075), 10 μg/mL Insulin-Transferrin-Selenium (Gibco, Waltham, MA, USA, 51300-044), 5.6 μg/mL linoleic acid (Sigma-Aldrich, St. Louis, MO, USA, L–9530), 0.1 μM dexamethasone (Sigma-Aldrich, D 2915) and 0.1 mM ascorbic acid-2 phosphate (Sigma-Aldrich) and 10 ng/mL TGFβ-3 (Novartis, Basel, Switzerland)), (ii) hPL-ChM (BM supplemented with 5% human platelet lysate (MultiPL’100i, Macopharma, Tourcoing, France, BC0190032), 40 U/mL Heparin-Natrium-25.000-ratiopharm^®^, 10 μg/mL insulin (Novo Nordisk, Bagsvaerd, Denmark), 0.1 mM ascorbic acid 2-phosphate). Pellets generated by centrifugation of 6 × 10^4^ cells/pellet (1500 rpm, 3 min) in 1.5 mL sterile conical micro tubes (Sarstedt, Nümbrecht, Germany) were cultured with 250 μL/tube of SFM-ChM or hPL-ChM for 21 or 28 days at 37 °C, 5% CO_2_, with media change twice/week. As a control, post-expanded NCs were also cultured in pellets with SFM-ChM [10]. There are few reports in the literature demonstrating that commercially available hPL preparations have been shown to negatively affect the matrix deposition of chondrocytes [53,54]. We thus carried out preliminary investigations to test the performance of hPL preparations (in comparison to fetal bovine serum) from different companies and selected MultiPL’100i from Macopharma for its capacity to enhance proliferation capacity of human nasoseptal chondrocytes while maintaining their good post-expansion cartilage-forming capacity.

#### 4.3.3. Culture of NCs in PEG Gels

PEG gel precursors, RGD peptide (cell attachment-site peptide for gel enrichment) and thrombin-activated factor XIII (FXIII, for PEG gel polymerization) were produced as previously described [24,25]. Briefly, to generate PEG precursors, 8-arm PEG-vinylsulfone (PEG-VS, 40 kDa MW; NOF) and a 1.2 molar excess of peptides (Bachem, purity of >95%) that contained either a factor XIII (FXIII) glutamine acceptor substrate sequence (Gln; H-NQEQVSPL-ERCG-NH_2_) or a matrix metalloproteinase (MMP)-degradable (*in italics*) FXIII lysine donor substrate (MMP_sensitive_-Lys; Ac-FKGG-*GPQGIWGQ*-ERCG-NH_2_) were dissolved in triethanolamine (TEA; pH 8.0). After functionalization for 2 h at 37 °C, the resulting 8-arm PEG-Gln and 8-arm PEG-MMP_sensitive_-Lys precursors were excessively dialyzed against pure water, lyophilized and stored at −20 °C. Hydrogels with the final concentration of 1.5% PEG were prepared by dissolving equimolar concentrations PEG precursors (8-arm PEG-Gln and 8-arm and PEG-MMP_sensitive_-Lys) in 50 mM Tris buffer pH 7.6 (Trizma^®^, Sigma-Aldrich, Ref. No. T1503) containing 50 mM CaCl_2_ (Fluka, Ref. No. 21101) and subsequent polymerization with thrombin-activated FXIII at 10 U/mL or at the indicated concentrations. *Rapidly* isolated NCs at a final concentration of 3.0 × 10^5^ cells/mL (6000 cells/20 μL gel) were added. This cell density was calculated by taking into consideration the cell yields expected from cartilage biopsies of 6 mm diameter [18] (i.e.,: 0.34 × 10^6^ ± 0.18 × 10^6^, N = 6), for a target cartilage lesion of an area of 5 cm^2^ and a thickness of 2 mm (corresponding to a final gel volume of 1 mL).

For human Platelet-Lysate(hPL)-enriched PEG gels, the PEG precursor solution was supplemented with hPL so that its final concentration in the hydrogels would be 5% *v*/*v*. Additionally, if not otherwise mentioned, RGD peptide was added at a final concentration of 50 μM. Alternatively, NC-PEG gels not containing hPL were prepared as controls, containing hSA instead (hSA-PEG). 

Hydrogel drops (20 μL, and for in vivo experiments 40 μL) were sandwiched between sterile hydrophobic glass microscopy slides separated by 1 mm high spacers and clamped with binder clips (see Supplementary Figure S1A). To prevent sedimentation of cells, the forming gels were slowly rotated at RT until the onset of gelation and then incubated at 37 °C for an additional 30 min to achieve complete cross-linking. The gels were then carefully detached from the slides using a sterile spatula. In order to ensure a good detachment of the disk-shaped gels when sandwiching them between the slides (Polysine^®^, Thermo Scientific, Waltham, MA, USA), a hydrophobic coating preparation with a siliconizing reagent for glass (SigmaCote^®^) was necessary.

NC-hPL enriched PEG gels were either cultured in vitro or used for the in vivo studies. Chondrogenic differentiation in vitro was performed by culturing NC-PEG gels in agarose(2%)-pre-coated 48-well plates (one gel construct/well) using hPL-ChM. Control NC-PEG gels (i.e., gels not containing hPL) were cultured in ChM containing 1.25 mg/mL human serum albumin (hSA) instead of hPL. Gels (named NC-hPL-PEG and NC-hSA-PEG, respectively) were cultured at 37 °C, 5% CO_2_ under static conditions for up to 28 days, with media change twice a week. Biochemical and histological analyses were conducted at specific culture times as described below. 

Gels were additionally prepared using different thrombin-activated FXIII concentrations and not containing the RGD motif, and analyzed as described below.

### 4.4. In Vivo Experiments

To acquire initial data on the in vivo performance of the NC-hPL-PEG gels, we used two ectopic models widely used to assess cartilage regenerative properties of cell-based grafts [28,63]. NC-hPL-PEG gels of a volume of 40 μL were implanted in the subcutaneous pockets of nude mice (CD-1 nude/nude; Charles River Laboratories, Ashland, OH, USA), alone (ectopic study) or after being directly injected in the defect created on osteochondral tissues (ectopic osteochondral study) as described below, to investigate their capacity to chondro-differentiate and integrate with surrounding articular tissues, respectively. All animal procedures were reviewed and approved by the Swiss Federal Veterinary Office (Permit No. BS 1797); animal experiments complied with the ARRIVE guidelines and were carried out according to the EU Directive 2010/63/EU.

#### 4.4.1. Ectopic Study

NC-hPL-PEG were generated as described above. Gels not containing NCs were used as negative controls. A total of 4 gels/mouse were implanted in the subcutaneous pocket of nude mice. The operation was performed with isoflurane (Attane Isoflurane; Provet AG, Lyssach, Switzerland) anesthesia and buprenorphine (Temgesic; Reckitt Benckiser AG, Wallisellen, Switzerland) analgesia, and animals were checked periodically. For each experiment, triplicate samples were generated (if not stated otherwise). After 4 weeks, mice were euthanized with CO_2_, and explants were assessed, as described below.

#### 4.4.2. Ectopic Osteochondral Study

Human osteochondral (OC) specimens were gained from 9 patients (5 males and 4 females, age 62.1 ± 9.8 years) undergoing total knee arthroplasty at the University Hospital of Basel, Switzerland after informed consent from patients and in accordance with the local ethical committee (University Hospital Basel; Prof. Dr. Kummer; approval date 26/03/2007 Ref Number 78/07). OC specimens were taken from macroscopically healthy areas (corresponding to cartilage lesions Grade 0 or 1 according to the International Cartilage Research Society) and fractioned in cylinders of approximately 0.6–0.8 cm diameter and 0.4 cm height. A defect with the size of 4 mm in diameter was generated by initially using a biopsy punch and a scalpel and finally a sharp Volkmann spoon, to complete the debridement until the calcified layer. NC-hPL-PEG gel (prepared as above described) was directly injected into the prepared lesions. Resulting constructs were then implanted directly (without pre-culture) in the subcutaneous pocket of nude mice (2 constructs/mouse). The gels and constructs prepared this way were immediately used for ectopic implantation. For each experiment, triplicate samples were generated (if not stated otherwise). After 4 weeks, mice were euthanized with CO_2_, and explants were assessed, as described below.

### 4.5. Analytical Methods

#### 4.5.1. Histology

Chondrogenic pellets and NC-PEG gels were fixed in 4% paraformaldehyde for 24 h. Paraffin-embedded samples were sectioned using the Microm HM355S microtome (Thermo Fisher Scientific) to 5 μm thickness. Alternatively, cryo-sectioning was performed after embedding in OCT^®^ (Tissue-Tek^®^ Optimal Cutting Temperature Compound, Sakura Finetek Europe, Alphen aan den Rijn, The Netherlands). After overnight incubation in OCT at 4 °C as a cryoprotectant [64], freezing in methyl butane and liquid nitrogen followed. The created blocks were then transferred onto dry ice and moved to a −80°C freezer for storage and kept there at least 24 h before cutting. Routine cryo-sectioning was performed on the frozen blocks using a cryostat at −20 °C. For each block, 7–8 μm sections were cut at the CM1950 cryostat (Leica Biosystems, Wetzlar, Germany) and collected on Superfrost Plus^®^ glass slides (Thermo Scientific). 

Safranin-O/Fast Green staining with hematoxylin (J.T. Baker) nuclear counterstaining was performed to analyze cartilage tissue formation. Bright field images were acquired with an inverted microscope (Nikon Eclipse Ti2). Safranin-O-/Fast-Green-stained slides were scored using the established Bern score [35]. Briefly, the Bern score has three rating parameters that each receive a score between 0 and 3. First, the intensity of Safranin O staining (0: no stain; 1: weak staining; 2: moderately even staining; 3: even dark stain), second, the distance between cells/amount of matrix produced (0: no space between cells; 1: cells < 1 cell-size apart; 2: cells approx. 1 cell-size apart; 3: >1 cell-size apart and extensive matrix) and third, the morphology of the cells (0: condensed/necrotic/pycnotic bodies; 1: spindle/fibrous; 2: mixed spindle/fibrous with rounded chondrogenic morphology; 3: majority rounded/chondrogenic). The three values are summed together resulting in a maximum possible Bern score of 9. 

Osteochondral tissues were decalcified using a 7% EDTA 30% sucrose solution (Sigma-Aldrich). The EDTA decalcification was supported by continuous stirring on the orbital shaker and carried out at a constant temperature of 37 °C. The solution was changed daily until bone was macroscopically soft, then EDTA was replaced with PBS and the samples were stored at 4 °C until further processing. Decalcified samples were then embedded in paraffin and sectioned (on slides of 5 μm of thickness) and stained for Safranin-O/Fast Green. Safranin-O-/Fast-Green-stained slides were scored using a newly developed system consisting of three categories, each with equal weight, with a possible minimum collective score of 0 and a maximum of 9. These scoring categories were: “integration with adjacent cartilage” (0: no integration, 1: <30% integration, 2: 30–60% integration, 3: >60% integration), “integration with calcified cartilage/subchondral bone” (0: no integration, 1: <30% integration, 2: 30–60% integration, 3: >60% integration), “quality of the cartilage distant from the margins” (0: absence of tissue; 1: presence of tissue not/weakly stained for Safranin-O and containing mainly fibroblastic cells; 2: presence, in scattered areas, of tissue intensely stained for Safranin-O and containing round cells; 3: presence of tissue uniformly stained for Safranin-O and containing round cells).

#### 4.5.2. Immunofluorescence

For collagen VI immunofluorescence staining after rapid isolation of the cells, NC-PEG gels were used directly after NC encapsulation (on “day 0”). They were immediately fixed in 4% paraformaldehyde for 24 h. Gels were then subjected to enzymatic epitope retrieval at 37 °C, first with 2 mg/mL hyaluronidase (Sigma-Aldrich, Ref. No. H3884) and then with 1 mg/mL pronase (Roche, Ref. No. 10 165 921001), blocked with 5% bovine serum albumin, and rabbit anti collagen VI (human and mouse specific, Abcam Ref. No. ab6588, 1:500 dilution) primary antibody was applied. Detection of immune-binding was performed with Alexa-Fluor-488-conjugated secondary antibody (goat anti-rabbit, Invitrogen, Ref. No. a11008, 1:200 dilution). DAPI was used as a nuclear counterstain. Stained sections were visualized using a confocal microscope (Zeiss LSM 710 Rocky) and images were processed with ImageJ.

#### 4.5.3. Immunohistochemistry

Immunohistochemical staining were performed manually or a with Ventana Discovery Ultra (Roche Diagnostics (Suisse) SA) automated slide stainer. Briefly, tissue sections were deparaffinized and rehydrated. Antigens were retrieved by a protease (Protease 3, ref. 760-2020, Ventana) digestion for 20 to 44 min at 37 °C, or by heat in Cell Conditioning buffer 1 (CC1, ref. 950-124, Ventana) at 95 °C for 64 min. Primary antibody was manually applied and incubated for 1 h at 37 °C. After washing, the HRP-polymer conjugated secondary antibody was incubated for 1 h at 37 °C. Detection was performed with the Vectastatin ABC kit (Vector Labs) or the Ventana DISCOVERY ChromoMap DAB (ref. 760-159, Ventana) detection kit. Afterwards, the slides were counterstained with hematoxylin. Sections were then dehydrated, cleared and mounted with permanent mounting and coverslips. Primary antibodies were used for type I collagen (ab137492, Abcam) at a dilution of 1:5000; type II collagen (63171, MP Biomedicals) of 1:1000; type VI collagen (ab6588, Abcam) of 1:500 and Ki67 (MA5-14520, Thermo Fischer Scientific) of 1:50. Undiluted, ready-to-use secondary antibodies used include anti-mouse polymer HRP (R&D Mouse IgG VisUCyte, ref. VC001-025) and anti-rabbit polymer HRP (Nichirei Histofine Simple Stain MAX PO (R), ref. 414141F).

#### 4.5.4. Quantification of Glycosaminoglycans and DNA

Chondrogenic pellets and NC-PEG gels were digested with 1 mg/mL protease K in 50 mM Tris with 1 mM EDTA, 1 mM iodoacetamide and 10 mg/mL pepstatin-A for 16 h at 56 °C. For glycosaminoglycan (GAG) quantification, the method of Barbosa et al. (2003) was used. Briefly, diluted or undiluted digested gels (depending on Safranin-O intensity) were incubated with 1 mL of dimethylmethylene blue assay (DMMB) solution (16 mg/L dimethylmethylene blue, 6 mM sodium formate, 200 mM GuHCL, pH 3.0) on a shaker at room temperature for 30 min. Precipitated DMMB-glycosaminoglycans (GAG) complexes were centrifuged and supernatants were discarded. Complexes were dissolved in decomplexion solution (4 M GuHCL, 50 mM Na-Acetate, 10% Propan-1-ol, pH 6.8) at 60 °C, absorption was measured at 656 nm, and GAG concentrations were calculated using a standard curve prepared with purified bovine chondroitin sulfate. DNA content was measured by using the CyQuant Cell Proliferation Assay Kit (Molecular Probes Inc., Eugene, OR, USA) according to the instructions of the manufacturer. Cell numbers in the in vitro NC-PEG gel experiments were calculated based on DNA content, estimating cell numbers in gels using the factor 7.7 pg DNA/cell as earlier described [36].

#### 4.5.5. Hydrogel Characterization by Rheometry

Hydrogel onset of gelation and stiffness was analyzed on a rheometer (MCR 301, Anton Paar) equipped with 20 mm plate–plate geometry (PP20, Anton Paar) at 37 °C in a humidified atmosphere. Gel mixtures were precisely loaded onto the center of the bottom plate. The upper plate was lowered to a measuring gap size of 0.2 mm, ensuring proper loading of the space between the plates and gel precursors; the dynamic oscillating measurement was then started. Measurements were recorded at the linear viscoelastic region of the hydrogels, at a constant angular frequency of 1 Hz and constant shear strain of 4%, as previously determined [48]. The gelation point was determined as the time when the storage modulus (G’) would exceed the loss modulus (G’’). The storage and loss modulus were reported at 30 min, when the equilibrium was reached.

#### 4.5.6. Statistical Analyses

All data are presented as mean values ±SD (standard deviation). Using the statistical analysis software GraphPad Prism, Mann–Whitney-U testing for non-parametric un-paired sample sets was performed if not otherwise mentioned. For comparison of more than two groups, (such as biochemical analyses of NC in PEG-gels with time in Figure 3B and for Bern scores in Table 1), non-parametric Kruskal–Wallis analyses followed by post hoc Dunn`s tests for multiple comparisons were performed. Unilateral *p* values < 0.05 were considered significant.

## Figures and Tables

**Table 1:** Total Bern score, Intensity of staining and Cell morphology of in vitro and in vivo NC-hPL-PEG constructs.

	Experimental Group (Culture/Incubation Time)	Tot Bern Score	Intensity of Staining	Cell Morphology
(a) in vitro	NC-hSA-PEG (control) (21 days)	1.9 ± 2.0	0.2 ± 0.3	0.5 ± 0.7
	NC-hPL-PEG (21 days)	6.5 ± 1.8 ***	1.6 ± 0.9 ***	2.2 ± 0.7 ***
	NC-hPL-PEG (28 days)	6.9 ± 1.6 ***	1.8 ± 0.6 ***	2.4 ± 0.6 ***
(b) in vivo	hPL-PEG (control) (28 days)	0	0	0
	NC-hPL-PEG (28 days)	5.7 ± 2.0 *	1.3 ± 0.6 **	2.0 ± 0.8

(a) In vitro NC-hPL-PEG constructs compared to in vitro NC-hSA-PEG constructs (control condition, 21 days). (b) In vivo NC-hPL-PEG (28 days) compared to in vitro NC-hPL-PEG (28 days). N = 23 explants (of 6 donors) in vitro, N = 16 explants (from 5 donors) in vivo. * *p* < 0.05, ** *p* < 0.002, *** *p* < 0.001 vs controls. Abbreviations: NC: nasoseptal chondrocytes, hSA: human serum albumin, hPL: human platelet lysate, PEG: polyethylene glycol.

**Table 2:** Histological scoring of NC-hPL-PEG in the ectopic osteochondral model.

	Total Score (Range: 0–9)	Integration with Cartilage (Range: 0–3)	Integration with Bone (Range: 0–3)	Quality of Tissue (Range: 0–3)
mean ± SD	5.3 ± 1.5	2.4 ± 0.8	1.8 ± 1.1	1.4 ± 1.0

Histological quality of the OC explants was assessed considering the following three categories: integration with cartilage (min: 0, max: 3), integration with bone (min: 0, max: 3) and quality in the tissue distant from the margins (min: 0, max: 3). Total *n* = 9 explants (from three donors).

## Data Availability

All data supporting the reported results can be available under request.

## Supplementary Materials

The following supporting information can be downloaded at: www.mdpi.com/article/10.3390/ijms23136900/s1.
