# Supplementary material for: In Vitro and Ectopic In Vivo Studies toward the Utilization of Rapidly Isolated Human Nasal Chondrocytes for Single-Stage Arthroscopic Cartilage Regeneration Therapy
Source: Int J Mol Sci. 2022 Jun 21;23(13):6900. doi: 10.3390/ijms23136900 (PMC9267018; doi:10.3390/ijms23136900)
Supplement: Supplementary file 1 [file ijms-23-06900-s001.zip › ijms-1758898-supplementary.pdf]

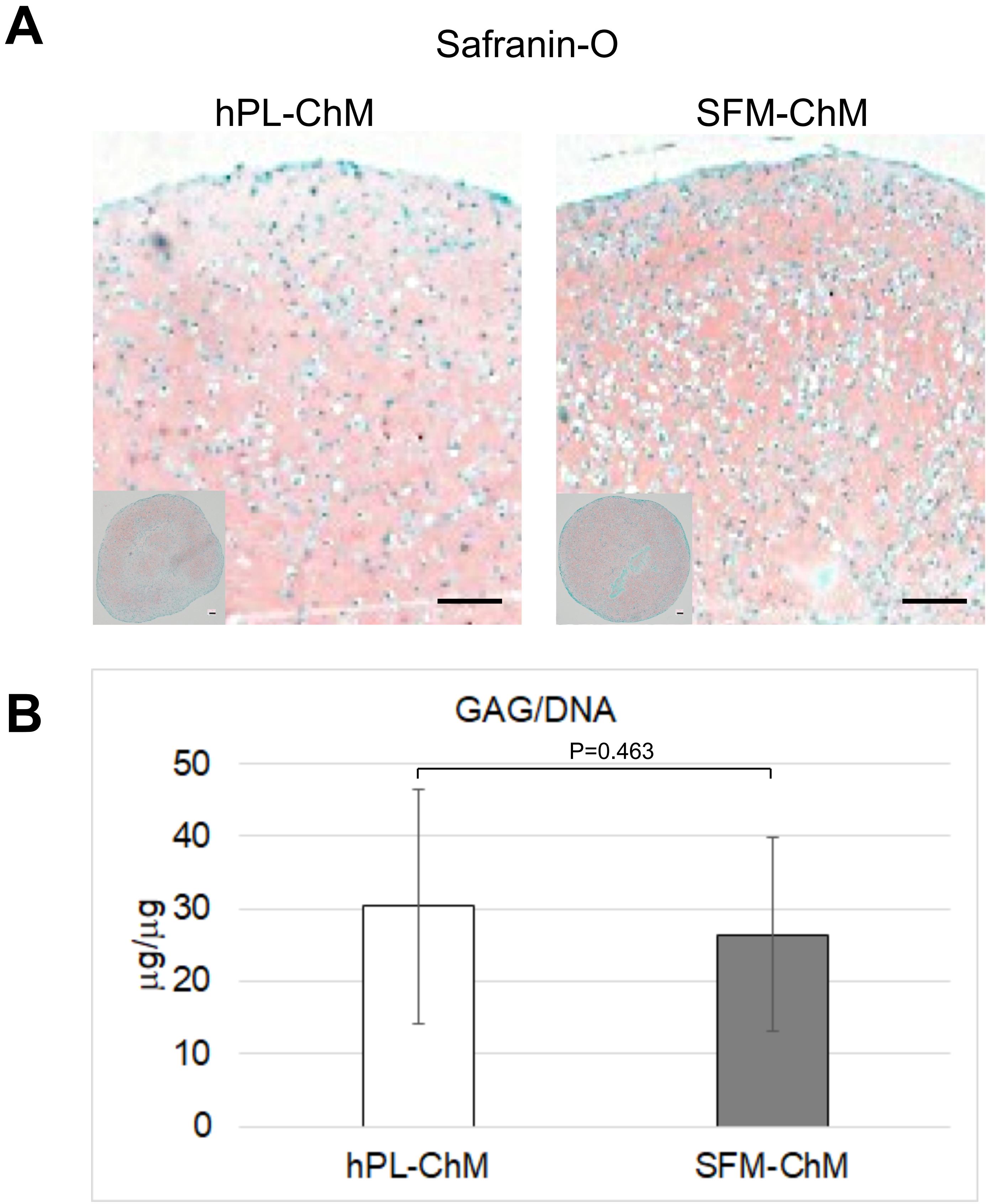

Supplementary Figure S1. Chondrogenic capacity of rapidly isolated, non-expanded nasal chondrocytes in SFM-ChM or hPL-ChM. Rapidly isolated P0 NCs were cultured in pellets in SFM-ChM or hPL-ChM (see “Materials and Methods” for the description of the composition) for 28 days. (A) Representative Safranin-O staining of resulting cartilaginous tissues. Scale bar = 100 µm. (B) Glycosaminoglycans/DNA (GAG/DNA) contents of generated cartilaginous tissues. Values are mean ± SD of results of samples generated with cells from 4 different donors.

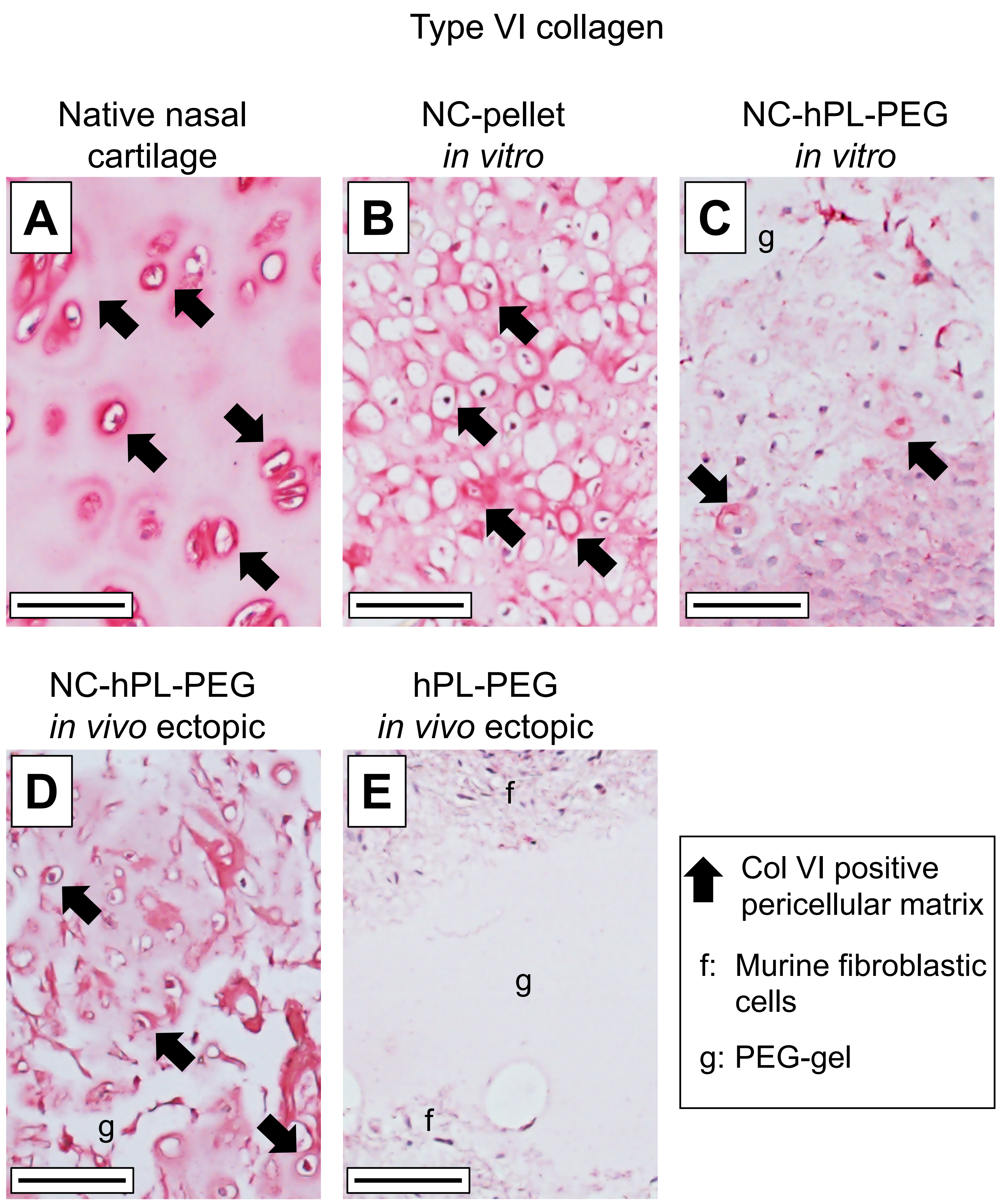

Supplementary Figure S2. Collagen type VI (Col VI) immunohistochemistry. Representative collagen (Col VI) staining of native cartilage sample (positive control, A), NC-pellets, (B) NC-hPL-PEG constructs cultured in vitro for 4 weeks (C), NC-hPL-PEG constructs (D) and hPL-PEG constructs (E, negative control) harvested from the mice (ectopic model) 4 weeks post-surgery. Scale bar = 100 µm.

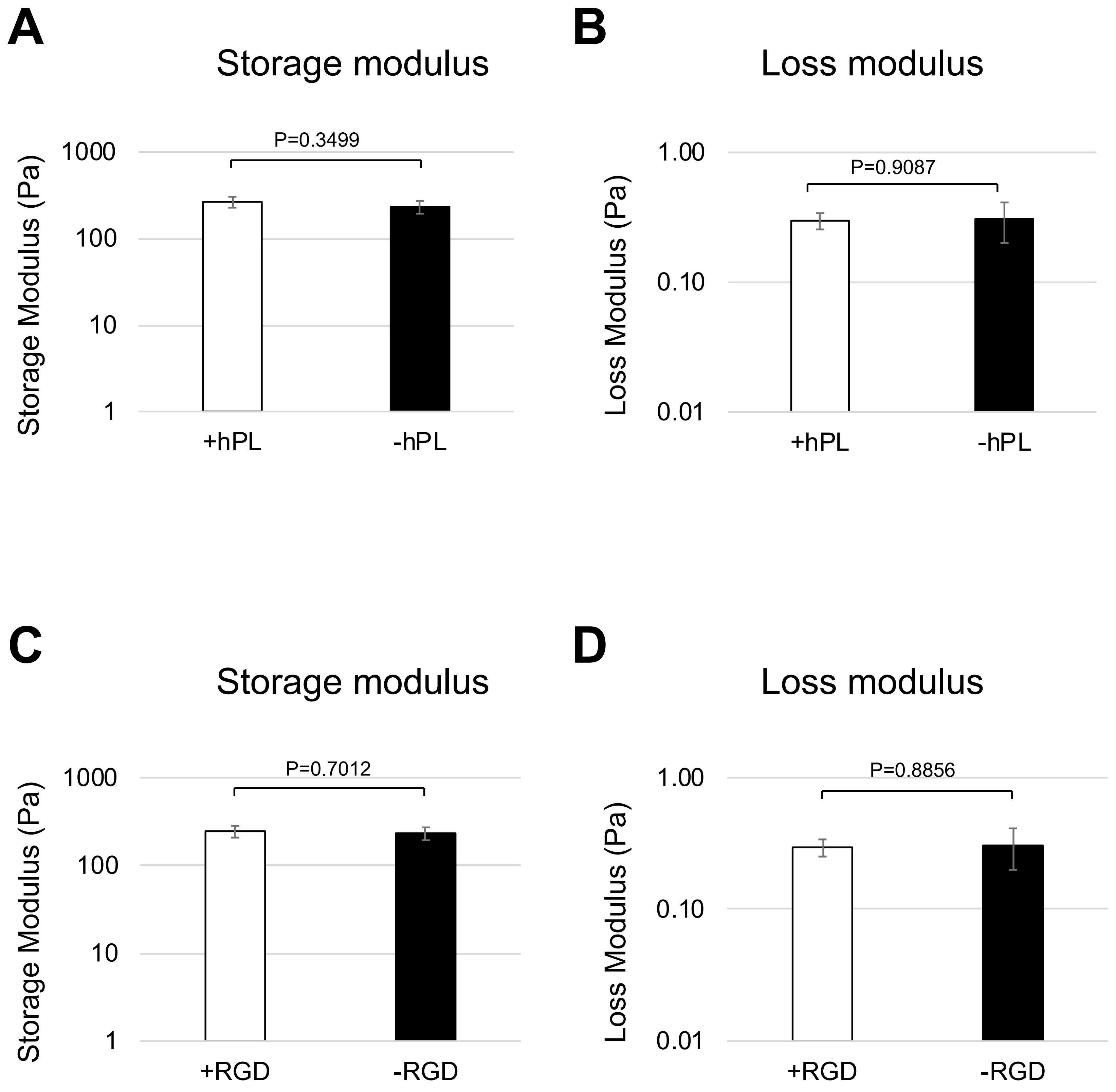

Supplementary Figure S3. Polyethylene glycol (PEG) gel stiffness in the presence or absence of human platelet lysate (hPL) or the RGD attached site peptides. Hydrogel stiffness was analyzed at 37 °C in a humidified atmosphere, storage and loss modulus were reported at 30 min when the equilibrium was reached. (A, C) Storage and (B, D) loss modulus did not significantly change by adding 5% v/v hPL to the 1.5 % PEG gel or by the presence or absence of RGD attachment site peptides in the 1.5 % PEG gel (see “Materials and Methods”).
